# High-Throughput Microdissection for Next-Generation Sequencing

**DOI:** 10.1371/journal.pone.0151775

**Published:** 2016-03-21

**Authors:** Avi Z. Rosenberg, Michael D. Armani, Patricia A. Fetsch, Liqiang Xi, Tina Thu Pham, Mark Raffeld, Yun Chen, Neil O’Flaherty, Rebecca Stussman, Adele R. Blackler, Qiang Du, Jeffrey C. Hanson, Mark J. Roth, Armando C. Filie, Michael H. Roh, Michael R. Emmert-Buck, Jason D. Hipp, Michael A. Tangrea

**Affiliations:** 1 Laboratory of Pathology, Center for Cancer Research, National Cancer Institute, National Institutes of Health, Bethesda, Maryland, United States of America; 2 Pathogenetics Unit, Laboratory of Pathology, Center for Cancer Research, National Cancer Institute, National Institutes of Health, Bethesda, Maryland, United States of America; 3 Cytopathology Section, Laboratory of Pathology, Center for Cancer Research, National Cancer Institute, National Institutes of Health, Bethesda, Maryland, United States of America; 4 Molecular Diagnostics Unit, Laboratory of Pathology, Center for Cancer Research, National Cancer Institute, National Institutes of Health, Bethesda, Maryland, United States of America; 5 Department of Mechanical Engineering, Johns Hopkins University, Baltimore, Maryland, United States of America; 6 Department of Pathology, University of Michigan, Ann Arbor, Michigan, United States of America; 7 Avoneaux Medical Institute, Oxford, Maryland, United States of America; 8 Alvin & Lois Lapidus Cancer Institute, Sinai Hospital, Baltimore, Maryland, United States of America; Johns Hopkins School of Medicine, UNITED STATES

## Abstract

Precision medicine promises to enhance patient treatment through the use of emerging molecular technologies, including genomics, transcriptomics, and proteomics. However, current tools in surgical pathology lack the capability to efficiently isolate specific cell populations in complex tissues/tumors, which can confound molecular results. Expression microdissection (xMD) is an immuno-based cell/subcellular isolation tool that procures targets of interest from a cytological or histological specimen. In this study, we demonstrate the accuracy and precision of xMD by rapidly isolating immunostained targets, including cytokeratin AE1/AE3, p53, and estrogen receptor (ER) positive cells and nuclei from tissue sections. Other targets procured included green fluorescent protein (GFP) expressing fibroblasts, *in situ* hybridization positive Epstein-Barr virus nuclei, and silver stained fungi. In order to assess the effect on molecular data, xMD was utilized to isolate specific targets from a mixed population of cells where the targets constituted only 5% of the sample. Target enrichment from this admixed cell population prior to next-generation sequencing (NGS) produced a minimum 13-fold increase in mutation allele frequency detection. These data suggest a role for xMD in a wide range of molecular pathology studies, as well as in the clinical workflow for samples where tumor cell enrichment is needed, or for those with a relative paucity of target cells.

## Introduction

**Fig 1:**
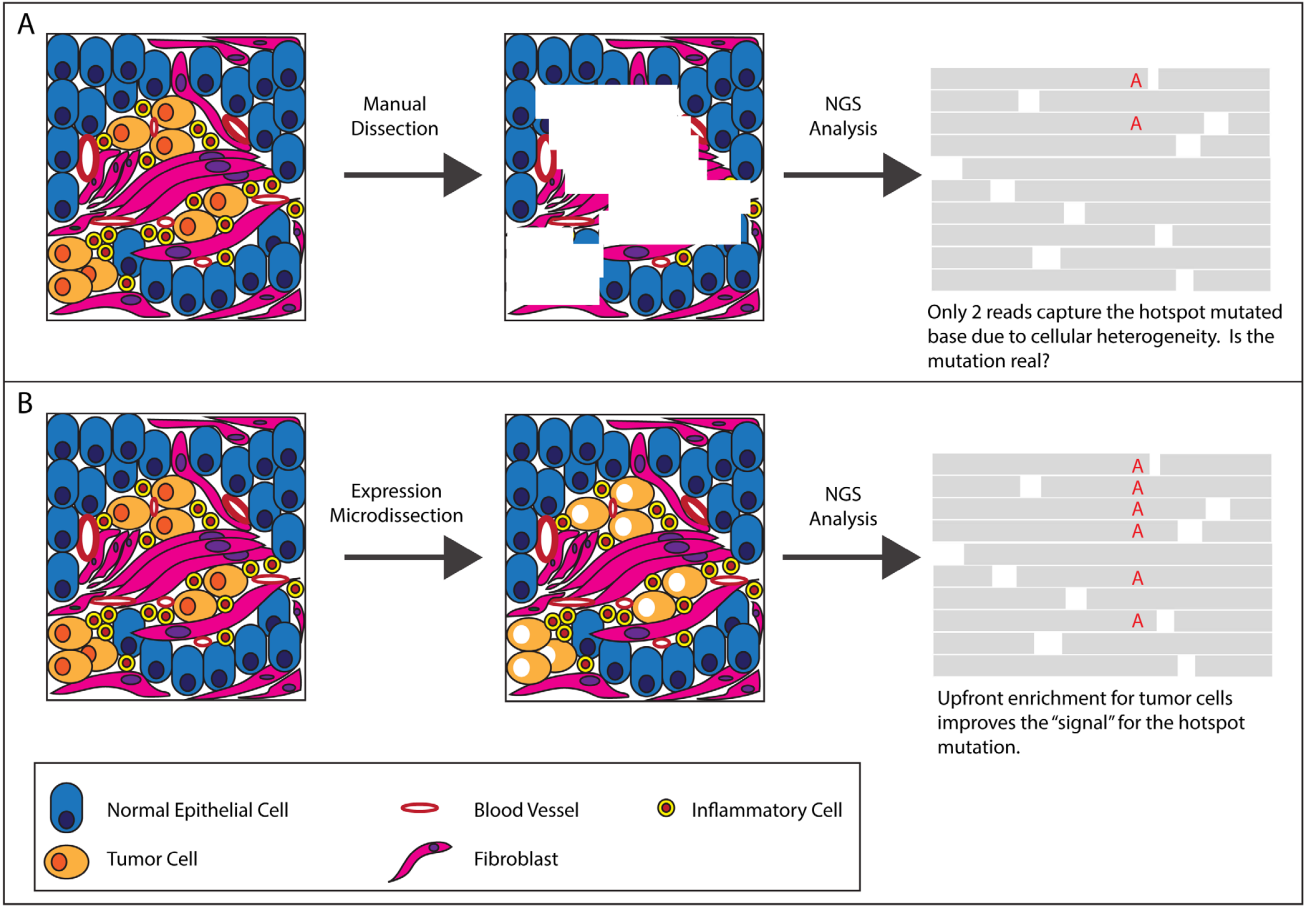
Potential impact of manual dissection versus xMD on NGS data.

Precision medicine builds on many decades of outstanding laboratory-based studies to benefit patients in the clinic. Today, identifying changes in DNA using next-generation sequencing (NGS) and other genomic methods is fueling this evolution. Many of the downstream analytic elements of the precision medicine workflow are well established. However, the current tools to isolate tumor cells from biospecimens, including solid tumors, are inadequate, being either too laborious or imprecise (Fig 1A). For example, laser-based microdissection technologies are generally impractical due to instrument cost, requirement for extensive pathologist time, and low-throughput. More crude, manual methods such as razor blade scrapes from histology slides are often acceptable for single gene mutation assays, but less so for complex NGS analyses of gene panels, and clearly inadequate for accurate expression-based (miRNA, mRNA, proteomic) measurements[1].

Expression microdissection (xMD) was invented to overcome limitations associated with commercial microdissection technologies for recovering cells from tissue sections (Fig 1B)[2, 3]. xMD leverages routine histochemical or immunohistochemical (IHC) staining with whole-slide irradiation to procure cellular or subcellular targets across entire slides. The stain absorbs the light energy, transiently heating the overlying ethylene vinyl acetate (EVA) polymer and bonding the targets to the film. Using various xMD prototype instruments, we have successfully isolated endothelium, epithelium, stromal components, and nuclei for downstream biomolecule analysis [2–8]. The combination of xMD precision (single cells or subcellular structures) and throughput enable investigators to rapidly collect sufficient target biomolecules for a variety of molecular analysis techniques, including PCR, mass spectrometry, array-based methods and immunoblotting[2–7]. We now report on the first flashlamp xMD application to NGS. Via xMD isolation of target neoplastic cells (melanoma and lung carcinoma cell lines) from an admixture, we demonstrate an improved signal-to-noise ratio in allele frequency for significant neoplasia-associated mutations.

## Results

### xMD Applications and Utility

As examples of precision medicine applications using the xMD flashlamp system, we isolated stained cells or nuclei from a spectrum of specimen types: formalin-fixed paraffin-embedded (FFPE) tissues, cytologic preparations, and cells cultured on slides.

**Fig 2:**
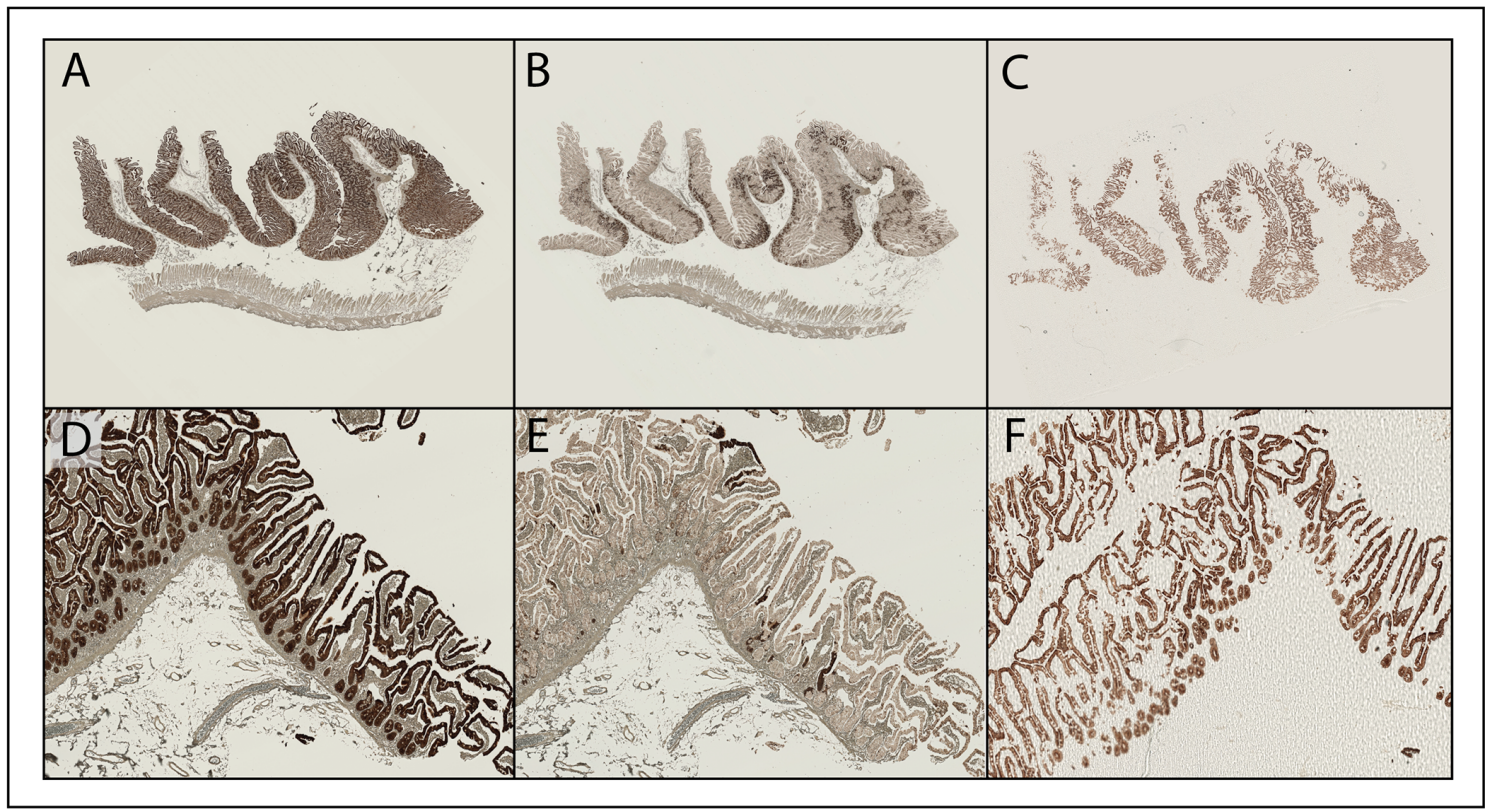
xMD application: Global epithelial microdissection. (A) a 1.25X digital image of the whole normal intestine specimen immunostained with cytokeratin AE1/AE3^+^ (B) a 1.25X digital image of the whole tissue following xMD, highlighting the degree of stained tissue procurement (C) a 1.25X digital image of the stained tissue bound to the xMD film (D-F) images of the before and after slide and film of the same specimen at higher (5x) magnification.

For a regional dissection, cytokeratin AE1/AE3-stained intestinal epithelium was isolated from the underlying submucosa, demonstrating the ability to capture a whole, contiguous feature in one step (Fig 2). This approach allows for the dissection of large regions of tissue from underlying and associated architecture to gain insight into the neoplastic transformation process for carcinomas. Using xMD, for example, one can compare neoplastic to benign epithelium to explore specific transformation events.

**Fig 3:**
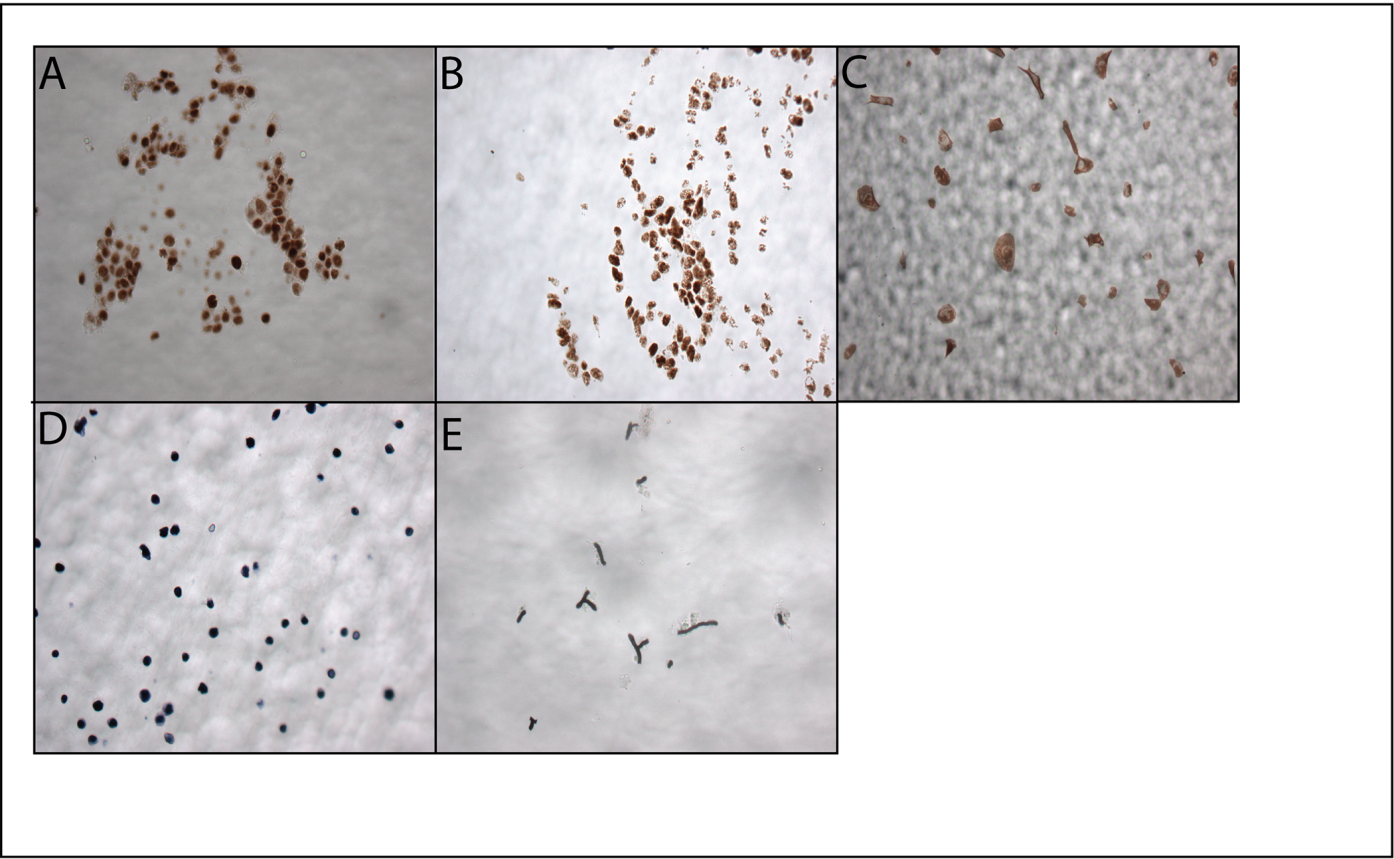
xMD applications: Nuclear and novel stain-based microdissection. (A) IHC stained p53^+^ nuclei from a section of metastatic colon carcinoma (B) IHC stained ER^+^ nuclei from a section of breast carcinoma (C) IHC stained GFP^+^ cells from a culture slide (D) EBER^+^ cells after EBV-EBER *in situ* hybridization (E) captured GMS^+^ *Aspergillus* fungal organisms.

Refined subcellular dissection is also possible with xMD[4]. Positively stained nuclei from a metastatic colon carcinoma and a primary breast carcinoma were efficiently dissected using nuclear immunostains,p53 and estrogen receptor (ER), respectively (Fig 3A and 3B). This approach allows for the capture of expression-specific nuclei from a heterogeneous microenvironment. Considering the extent of cell diversity present within a typical specimen (i.e, infiltrating leukocytes, fibroblasts, vasculature), specific enrichment of nuclear subpopulations allows for the identification of the signature and/or driver mutations responsible for a particular target of interest.

Applying xMD to cell biology studies, we employed anti-green fluorescent protein (GFP) immunostaining to rapidly isolate GFP expressing fibroblasts from a culture slide containing a mixed cell population of GFP^+^ fibroblasts and non-GFP expressing epithelial cells (Fig 3C). The novel ability to procure specific cells based on their expression of GFP will allow for the evaluation of cell type specific changes in a variety of experimental model systems.

To demonstrate the ability of xMD to procure cells based on mRNA targeting instead of proteins, we used *in situ* hybridization (ISH) staining of Epstein-Barr encoding region (EBER) mRNA to successfully dissect EBER^+^ lymphocytes from a FFPE sample (Fig 3D). In the setting where either a suitable antibody is not available or where targeting is based on RNA, not protein, the use of *in situ* hybridization is a useful alternative strategy for xMD.

In addition to targeting transcripts by ISH or proteins by IHC, histochemical stains that rely on other chemical properties can also be used to guide the dissection process. For example, Gomori methenamine silver (GMS) stained fungi [9] from a FFPE tissue section were rapidly isolated with the xMD system (Fig 3E). Infectious agents, such as fungi, are sometimes only present in small numbers making molecular identification difficult. Thus, the ability to specifically isolate fungi from complex matrices may prove valuable for future clinical and research applications.

Taken together, these examples demonstrate that xMD is a dynamic system with the ability to target a range of stained cellular and subcellular targets.

### DNA Analysis of Dissected Cell Populations

As an example of xMD utility, we isolated scattered cells of interest from a mixed sample, where the target cells constituted ~5% of the total cellularity. Specifically, cytospin-prepared slides with an admixture containing a majority of Burkitt lymphoma cells (ST486, *KRAS* and *BRAF* wild-type) and either a lung carcinoma cell line (A549, *KRAS*^*+*^ homozygous mutation) or melanoma cell line (UACC.62, *BRAF*^*+*^ heterozygous mutation) in a 20:1 ratio were generated and used in all subsequent experiments. The cytospins were immunostained with cytoplasmic or nuclear markers typically used in clinical practice: cytokeratin AE1/AE3 for carcinomas, melanoma-associated antigen recognized by T cells (MART-1) for melanoma, and thyroid transcription factor 1 (TTF1) for lung carcinoma.

#### Pyrosequencing

Utilizing the heterogeneous cytospins, *KRAS* and *BRAF* gene mutational status from xMD-dissected cancer cells was compared to manual macrodissected cytospin slides by pyrosequencing. The TTF1^+^ xMD procured lung carcinoma cells demonstrated a 5-8-fold increase in KRAS mutation percentage in comparison to the whole cytospin macrodissection (46–76% vs. 9%). Likewise, the MART-1^+^ xMD procured melanoma cells demonstrated an approximate 2-fold *BRAF* mutation enrichment via pyrosequencing (14–33% vs. 8–9%). We thus show substantial enrichment via xMD for tumor cell line specific mutations using low-plex techniques.

#### NGS

**Fig 4:**
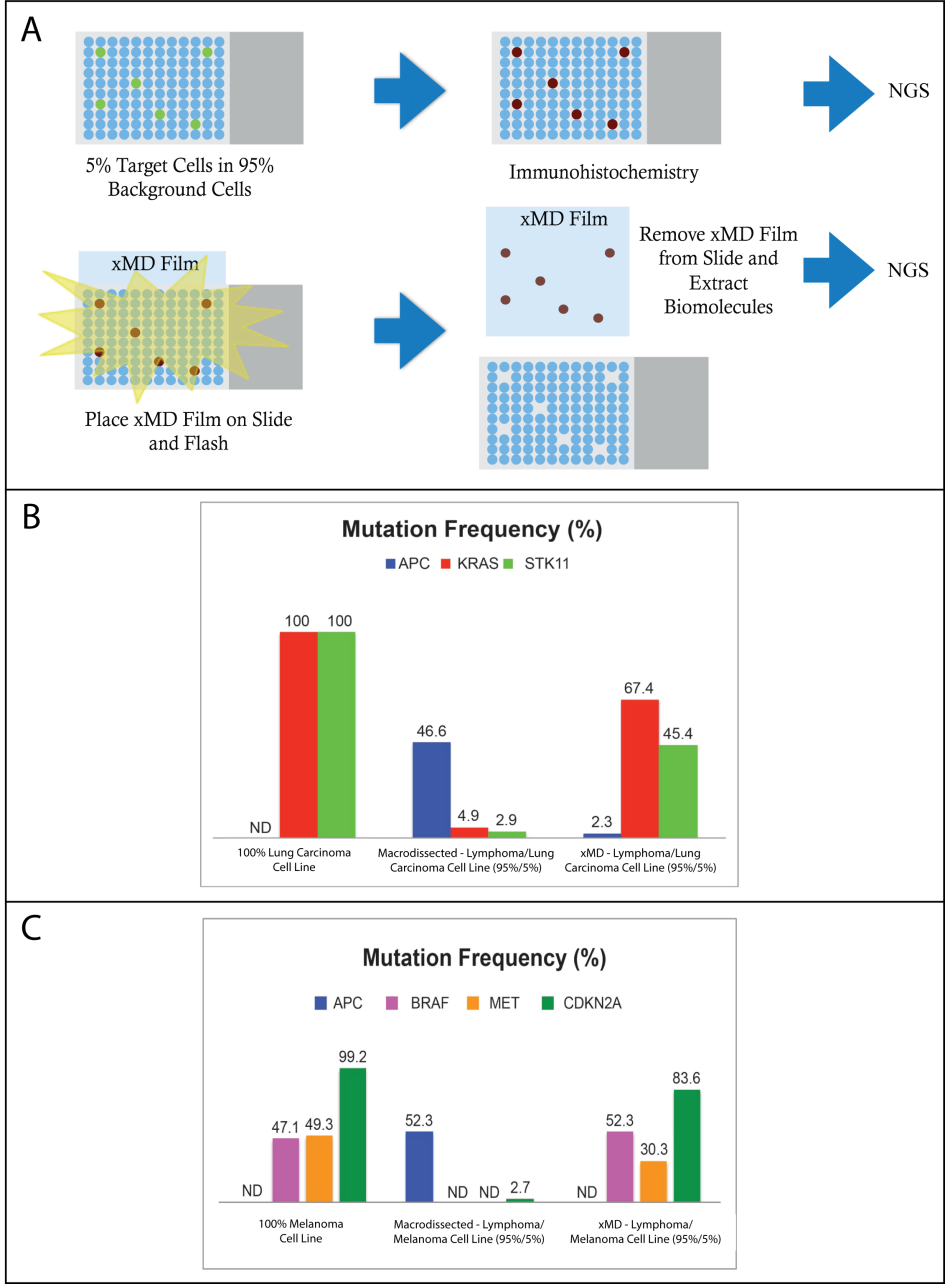
xMD improves depth of NGS coverage in admixed cell cytospins. (A) Schematic image of the NGS workflow. xMD isolated targets were compared to manual macrodissection via NGS (B) Select variant evaluation of the 95% lymphoma (ST486)/5% lung carcinoma (A549) cell line specimens comparing manual macrodissection to xMD enrichment (C) Select variant evaluation of the 95% lymphoma (ST486)/5% melanoma (UACC.62) cell line specimens comparing manual macrodissection to xMD enrichment. ND = not detectable.

Next, we compared the DNA from pure cell lines (ST486, A549, and UACC.62) versus the manual macrodissected and xMD-dissected heterogeneous cytospin preparations using the Ion AmpliSeq Cancer Hotspot Panel (V2) on the IonTorrent PGM system (Fig 4A). Using the 50 cancer-gene panel we confirmed mutations/variants unique to each of the three cells lines, which included: A549 lung carcinoma cell line–homozygous *KRAS* mutation (c.34G>A (p.Gly12Ser)) and homozygous STK11 mutation (c.109C>T (p.Gln37*))[10]; UACC.62 melanoma cell line–heterozygous *BRAF* (c.1799T>A (p.Val600Glu))[10], homozygous *CDKN2A* (c.242C>T (p.Pro81Leu)), and heterozygous *MET* (c.2975C>T (p.Thr992Ile)) (Table 1, S1 and S2 Tables). In addition, a heterozygous single-nucleotide polymorphism (SNP, dbSNP# rs1801166) in *APC* was found in the ST486 Burkitt lymphoma cell line (c.3949G>C (p.Glu1317Gln)). Interestingly, the cytokeratin AE1/AE3-stained xMD-procured lung carcinoma cells showed a 13-fold increase in *KRAS* mutation frequency (%) in comparison to the macrodissected heterogeneous cytospin (67.4% vs. 4.9%) and a 15-fold increase in the *STK11* mutation frequency (%) (45.4% vs. 2.9%) (Fig 4B). The MART-1^+^ xMD-selected melanoma cells demonstrated a substantial increase in BRAF and MET mutation frequency compared to the macrodissction of the admixture. In the xMD-derived specimen *BRAF* and *MET* mutation allele frequencies were 52.3% and 30.3%, respectively, and were undetectable in the macrodissected admixture (Fig 4C). In addition, a third melanoma cell line-specific mutation, CDKN2A, showed an approximate 30-fold mutation enrichment in the xMD sample (83.6% vs. 2.7%). Conversely, the Burkitt lymphoma cell line-specific SNP, APC, demonstrated a 20-fold or greater suppression following xMD (lymphoma/lung carcinoma cell line—46.6% vs. 2.3; lymphoma/melanoma cell line—52.3% vs. undetectable). Additional gene variants were identified in the xMD specimens and overall showed a similar trend with enrichment for variants in the target population and suppression of variants in the background Burkitt lymphoma cells, supporting the specificity of the technology on a clinically relevant NGS platform (S1 and S2 Tables).

**Table 1:** xMD impact on NGS data quality for highly penetrant disease variants.

Sample	% of ≥Q20 Bases	Mapped Reads	Mean Depth	Gene	Frequency (%)
100% ST486	88.3	551,287	2,183	APC^1^	51.5
				TP53^2^	57.8
				TP53^3^	51.7
				TP53^4^	48.9
100% A549	91.4	823,069	3,794	KRAS^5^	100
				STK11^6^	100
				TP53^2^	96.7
100% UACC.62	86.2	374,503	1,209	BRAF^7^	47.1
				CDKN2A^8^	99.2
				MET^9^	49.3
5% UACC.62-95% ST486 (Macrodissected)	86.9	661,917	2,872	APC^1^	52.3
				BRAF^7^	ND
				CDKN2A^8^	2.7
				MET^9^	ND
				TP53^2^	59.4
				TP53^3^	47.9
				TP53^4^	49
5% UACC.62-95% ST486 (xMD)	87.5	544,533	2,301	APC^1^	ND
				BRAF^7^	52.3
				CDKN2A^8^	83.6
				MET^9^	30.3
				TP53^2^	7.1
				TP53^3^	15.9
				TP53^4^	12.8
5% A549-95% ST486 (Macrodissected)	87.1	985,460	4,194	APC^1^	46.6
				KRAS^5^	4.9
				STK11^6^	2.9
				TP53^2^	56
				TP53^3^	45.1
				TP53^4^	43.8
5% A549-95% ST486 (xMD)	87.5	543,246	1,988	APC^1^	2.3
				KRAS^5^	67.4
				STK11^6^	45.4
				TP53^2^	78.3
				TP53^3^	5.2
				TP53^4^	7.3

Ion Torrent PGM Data of macrodissected versus xMD-dissected cytospins. The quality of the amplicon libraries is reflected in the percent ≥Q20 base, mapped reads and read depth. The gene and gene variants are listed. Frequency represents the percentage of reads for a particular amplicon with the variant of interest relative to the wild type variants.

^1^c.3949G>C (p.Glu1317Gln)

^2^c.215C>G (p.Pro72Arg)

^3^c.473G>A (p.Arg158His)

^4^c.715A>G (p.Asn239Asp)

^5^c.34G>A (p.Gly12Ser)

^6^c.109C>T (p.Gln37*)

^7^c.1799T>A (p.Val600Glu)

^8^c.242C>T (p.Pro81Leu)

^9^c.2975C>T (p.Thr992Ile)

ND = Not Detectable.

## Discussion

xMD is a high-throughput microdissection technology that can provide enriched samples for precise DNA sequencing-based studies. For the first time this microdissection technique was utilized in conjunction with NGS to show the value of isolating specific cell populations from a heterogeneous environment, similar to flow cytometry analysis for liquid tumors. New advances in understanding basic tumor biology and genetics have the potential to improve the lives of patients diagnosed with cancer through better diagnostic methods, prognostic capabilities, and treatment options. In particular, the ability to select the best therapy based on the molecular profile of a patient’s tumor, i.e. precision medicine, represents an exciting evolution in cancer care.

Manual macrodissection and laser-based microdissection technologies selectively recover histologic regions or cells from cytology preparations and tissue sections. Yet, due to the high-cost and time-intensiveness of laser dissection, coarse macrodissection is the most commonly used tool for laboratory-based studies and clinical applications. Through the evolution of xMD, we have achieved procurement rates of thousands of cells/second in a completely automated fashion and without the need for human direction[3–5]. Today, the low-energy flashlamp xMD system dissects all targeted cells (or organelles) in a histological section in less than 30 seconds, whether there are 100 targets or 100,000 targets. This capability makes xMD a practical approach in a busy molecular diagnostics laboratory where it is unrealistic for a pathologist to spend extensive amounts of time dissecting tumor cells from patient specimens under microscopic visualization.

The detection of mutations in cytologic and histologic preparations is dependent on the analytic sensitivity of the molecular diagnostic technique. Absence of a mutation may occur in two scenarios: (a) a true negative in which the tumor cells being interrogated do not harbor the mutation; and/or (b) a false negative where the molecular technique does not have the sensitivity to detect low frequency mutant allele(s) in tumor cells due to the dilutional effect of the wild type alleles from the abundant benign cellular elements in the background [11, 12]. With the admixed sample tested, the latter scenario was observed, where the genomic signature of the low frequency target cells (5%) was masked by the abundant background cells (ST486) in the macrodissected/whole sample input. However, xMD overcame this significant limitation and the variant profile of the target cells matched that of the pure cell line input with allele frequencies approaching those observed in the pure melanoma and lung cancer cell lines. Furthermore, an APC variant found in the background Burkitt lymphoma cell line (ST486) was suppressed in the xMD specimen, supporting the improved specificity of the xMD technique. These data demonstrate an important utility of xMD to potentially reduce the number of false negative sequencing results due to limited target cell input, which may be relevant to clinical applications.

Pathologists have at their disposal a large number of immunostains that can selectively highlight malignant cells from background. Leveraging these immunostains with xMD will enable analysis of samples that otherwise would be suboptimal for molecular diagnostics. For example cytopathologists/molecular pathologists may find xMD useful for selective enrichment of tumor cells from a cytologic specimen with low abundance of tumor cells in a background of primarily benign cellular contaminants.

In summary, we describe a flashlamp xMD system that facilitates rapid isolation of specific targets from a heterogeneous environment for a wide range of research and clinical NGS applications.

## Materials and Methods

### Tissue and Cytologic Samples

Anonymized tissue specimens were used in all experiments and obtained under a National Institutes of Health, Office of Human Subject Research exemption. All samples were sectioned to 4–5 micron thickness onto standard charged 1 inch x 3 inch glass microscope slides and air-dried. The cell lines used in the study included: ST486 cell line (ATCC) and the UACC.62 and A549 cell lines (NCI Anti-cancer Drug Screen, an in-house cell repository). Cell lines were trypsinized and washed to generate air-dried monolayer cytospins. All slides were immediately stored in a desiccator with desiccant (DRIE-RITE) until staining. Co-culture slides containing GFP-NIH3T3 fibroblasts (5%) and 4T1 epithelial cells (95%) were grown for 3 days after seeding and processed for staining.

### Immunohistochemical Staining

Diff Quik and H&E stains were performed on cytospin and tissue samples to assess morphology and adequacy. Immunohistochemistry staining was completed on either the Ventana Benchmark or Ultra Autostainer systems. For the Ventana Benchmark system, tissue slides were baked at 60°C for 20 minutes. After baking, tissues were placed in xylenes for 10 minutes and rehydrated through graded alcohols and placed in water prior to microwave antigen retrieval [13]. For cytospins, the slides were fixed in neutral buffered formalin for 40 minutes and then placed in water prior to microwave antigen retrieval. Co-culture slides (GFP-NIH3T3 fibroblasts and 4T1 epithelial cells) were formalin fixed for 10 minutes and subsequently permeabilized by Triton-X100 (0.5% in PBS) for 5 minutes. Slides were placed in a staining rack within a Tissue Tek staining dish, filled with 250ml of 1x Citra Plus (BioGenex), and covered loosely. The slides were microwaved for 5 minutes at high power (Sanyo, 1200 Watts) until boiling. The buffer level was checked and diH_2_O was added, if necessary. The tissue sections were kept moist throughout the procedure. The slides were then microwaved for 15 minutes at power level 3 and the buffer level was inspected halfway through the 15 minutes and diH_2_O added if needed. The slides were allowed to cool down in the microwave for an additional 20 minutes. Finally, the slides were rinsed with diH_2_O.

Following antigen retrieval, the slides were incubated at room temperature for 2 hours with primary antibody and detection was completed using the iView kit and DAB chromogen with no counterstain. The primary antibodies used for immunohistochemistry included; cytokeratin AE1/AE3 antibodies (1:100, DAKO), MART-1 (1:400, Cell Marque), TTF-1 (1:10, Novocastra/Leica Microsystems), p53 (1:1000, DAKO), ER (1:40, Novocastra/Leica Microsystems), and GFP (1:500, Abcam). After DAB, slides were rinsed in water and dehydrated through graded alcohols to xylenes (only about 10 dips in each xylenes bath to minimize exposure). When preparing slides for dissection, counterstains were avoided and non-specific staining limited to increase the contrast between the stained targets and background. Following IHC, the stained slides were air-dried and immediately stored in a desiccant chamber until dissection.

### EBER Staining

*In situ* hybridization for EBV-encoded RNA (EBER) was conducted on FFPE sections using probe (760–1209) EBER 1 DNP (Ventana) that binds to EBV encoded RNA1 on an automated stainer (Ventana-Benchmark XT). Visualization was achieved using the ISH iView system with Alk-Phosphatase and NBT/BCIP substrate with no counterstain.

### xMD Components

The xMD system consists of a vacuum system (FoodSaver), a flashlamp device (SensEpil, HomeSkinovations), and an ethylene vinyl acetate (EVA) polymer film (9702, 3M). Pint-sized bags (FoodSaver) were used to ensure close physical contact of the EVA film with the stained sample. The particular EVA polymer utilized in the study exhibited a melting point near 95°C (95°C—115°C) and had a thickness of 75 microns. A water-dampened 2.5 mm thick, white blotting filter paper (Bio-Rad) was placed between the flashlamp and the vacuum-sealed sample. The flashlamp provided a broad-spectrum (350-1200nm wavelength) high intensity light over the entire tissue slide. With a 1 x 2 inch window, the device provided between 2.8 J/cm^2^ and 4.8 J/cm^2^ of energy using a xenon flash tube with a 20 to 500 microsecond flash period.

### xMD Process

The xMD process was performed as previously described [5]. The number of flashes and power setting were determined for each sample type and varied depending on the stain intensity. For the flashlamp used in our studies, the intensity was typically set to level 2 or 3 and pulsed 3 to 5 times for each dissection with an overall operation time of less than 30 seconds per sample. Note that the operator should employ proper eye safety measures during the process to protect against reflected light [14]. Following irradiation, the vacuum bag was opened and the film containing the captured targets was carefully lifted off the slide. Finally, the film was placed in a clean microcentrifuge or PCR tube where the biomolecules were extracted by an appropriate lysis buffer.

### Imaging

To capture EVA film images, films were placed on glass slides and wetted on both sides with 100% ethanol or mounted with ProLong Antifade (ThermoFisher), and then coverslipped. High-resolution images were captured using an Olympus BX41 microscope outfitted with an Olympus Q-Color3 camera or a Hamamtsu Nanozoomer HT 2.0 slide scanner.

### DNA Isolation

All manual macrodissections were performed with a razor blade. DNA was isolated using the QIAamp DNA micro kit (Qiagen). Briefly, sufficient digestion buffer (300–500 μL total volume) with proteinase K was used to entirely cover the xMD film in a 1.5 mL microcentrifuge tube. Following overnight proteinase K digestion at 56°C an additional 1 hour incubation at 90 °C was performed to inactivate the enzyme. DNA was isolated and eluted in 20μL of buffer according to the manufacturer's instructions.

### Mutational Testing

The mutation tests for BRAF c.1799T>A (p.V600E) and KRAS c.34G>A (p.G12S) were performed using target-specific COLD-PCR [15] followed by pyrosequencing on a PyroMark Q24 instrument (Qiagen). Briefly, PCR reactions were conducted in a total volume of 25 μL containing 5 μL genomic DNA template, 200 nM of each forward and reverse primers, and 12.5 μL 2x HotStarTaq Master Mix (Qiagen) under the conditions described previously[16, 17]. For the pyrosequencing reactions, 10 μL of PCR product was immobilized on streptavidin-coated Sepharose beads (GE Healthcare) and processed according to the manufacturer’s instructions.

### Targeted Amplicon Library Preparation

Library preparation was carried out using the Ion AmpliSeq Cancer Hotspot, Panel V2 and the Ion AmpliSeq Library Kit 2.0, according to the directions provided in the corresponding Ion Torrent User Guide. The Ampliseq Panel includes 207 primer sets covering ~2,800 COSMIC hotspot mutations in 50 cancer-related genes. Briefly, individual DNA samples were subjected to multiplex PCR in a 96-well plate and library cleanup, followed by ligation to Ion Xpress Barcode Adapters 1–16. Each barcoded library concentration was determined by qPCR with Ion Library TaqMan Quantitation Kit (Life Technologies) on a ViiA 7 Real Time PCR System.

### Emulsion PCR and Sequencing

Each sequencing template, consisting of 4 barcoded libraries with equal concentration (5 μL at 20 pM concentration each), was amplified by emulsion PCR using the Ion PGM Template OT2 200 Kit on a OneTouch 2 instrument, following the instructions in the Ion Torrent User Guide. Following Emulsion PCR, the templated Ion Sphere particles (ISPs) were recovered and 1.0 μL aliquots of both enriched and unenriched ISPs were stained with SYBR Green Nucleic Acid Gel Stain (Life Technologies) and assessed for ISP quality and quantity on a Guava easyCyte 5 Flow Cytometer (Millipore). Approximately 20 million enriched ISPs were loaded into a 316 v2 chip and sequencing was performed on the PGM with the Ion PGM Sequencing 200 Kit v2.

### Data Analysis

Mutation analysis was carried out with Torrent variantCaller (v4.0.6), and confirmed using Integrative Genomics Viewer (IGV) (http://www.broadinstitute.org/software/igv/home). Ion Reporter 4.0 was used for variant annotation and classification. Variants with a frequency ≥30 were used in the assessment of xMD-related enrichment/depletion.

### Data Sharing

The raw.fastq files are available for download and analysis at the European Nucleotide Archive (ENA) under study accession number (http://www.ebi.ac.uk/ena/data/view/PRJEB12687). The processed data output including all variants identified with Torrent variantCaller is presented in S3 Table.

## Supporting Information

S1 Table Genomic variants identified in the lung carcinoma cell line. A total of sixteen (16) hotspot or novel variants were identified in parental A549 lung carcinoma cell line and ST486 Burkitt lymphoma cell lines (allele frequency ≥ 30%). Seven (7) variants showed enrichment in the A549 xMD sample, six (6) variants that were ST486-derived were suppressed in the A549 xMD sample. Two (2) variants demonstrated equal frequencies in both cell lines. One variant, although present in both parent cell lines, was not detected in the xMD specimen.
(DOCX)

S2 Table Genomic variants identified in the melanoma cell line. A total of sixteen (16) hotspot or novel variants were identified in parental UACC.62 melanoma cell line or ST486 Burkitt lymphoma cell line (allele frequency ≥ 30%). Five (5) variants showed enrichment in the UACC.62 xMD sample, nine (9) variants that were ST486-derived were suppressed in the UACC.62 xMD sample. Two (2) variants demonstrated equal frequencies in both cell lines.
(DOCX)

S3 Table All variants identified from analysis of raw data (Torrent variantCaller 4.0). All the variants identified are listed. Each sample is presented in a separate tab. Allele frequency, quality and coverage data is included.
(XLSX)
