# Supplementary material for: High-Throughput Microdissection for Next-Generation Sequencing
Source: PLoS One. 2016 Mar 21;11(3):e0151775. doi: 10.1371/journal.pone.0151775 (PMC4801357; doi:10.1371/journal.pone.0151775)
Supplement: S1 Table — A total of sixteen (16) hotspot or novel variants were identified in parental A549 lung carcinoma cell line and ST486 Burkitt lymphoma cell lines (allele frequency ≥ 30%). Seven (7) variants showed enrichment in the A549 xMD sample, six (6) variants that were ST486-derived were suppressed in the A549 xMD sample. Two (2) variants demonstrated equal frequencies in both cell lines. One variant, although present in both parent cell lines, was not detected in the xMD specimen. (DOCX) [file pone.0151775.s001.docx]

| Variant Status | Chromosome | Position | Gene | Ref | Variant | ST486 | A549 | A549-M | A549-X | Variant type | Source of Variant |
| --- | --- | --- | --- | --- | --- | --- | --- | --- | --- | --- | --- |
| xMD-Enriched | 2 | 209113192 | IDH1 | G | A | 0 | 34.9 | 2.8 | 25.6 | Hotspot | A549 |
|  | 3 | 178917005 | PIK3CA | A | G | 0 | 98.9 | 0 | 57.7 | Novel | A549 |
|  | 10 | 43615633 | RET | C | G | 0 | 65.2 | 3.6 | 50.2 | Novel | A549 |
|  | 10 | 43613843 | RET | G | T | 50.9 | 100 | 53.3 | 78.7 | Novel | ST486/A549 |
|  | 12 | 25398285 | KRAS | C | T | 0 | 100 | 4.9 | 67.4 | Hotspot | A549 |
|  | 17 | 7579472 | TP53 | G | C | 57.8 | 96.7 | 56 | 78.3 | Novel | ST486/A549 |
|  | 19 | 1207021 | STK11 | C | T | 0 | 100 | 2.9 | 45.4 | Hotspot | A549 |
| xMD-Depleted | 2 | 212812097 | ERBB4 | T | C | 50 | 0 | 50 | 17.9 | Novel | ST486 |
|  | 5 | 112175240 | APC | G | C | 51.5 | 0 | 46.6 | 2.3 | Hotspot | ST486 |
|  | 11 | 534242 | HRAS | A | G | 49.8 | 31.8 | 46.4 | 32.7 | Hotspot | ST486/A549 |
|  | 17 | 7578457 | TP53 | C | T | 51.7 | 0 | 45.1 | 5.2 | Hotspot | ST486 |
|  | 17 | 7577566 | TP53 | T | C | 48.9 | 0 | 43.8 | 7.3 | Hotspot | ST486 |
|  | 18 | 48586344 | SMAD4 | C | T | 47.1 | 0 | 46.3 | 10.6 | Novel | ST486 |
| Unchanged | 5 | 149433597 | CSF1R | G | A | 97.7 | 98.2 | 98 | 82.4 | Novel | ST486/A549 |
|  | 5 | 112175770 | APC | G | A | 45.2 | 33.5 | 47.5 | 52.3 | Novel | ST486/A549 |
| xMD-Not Detected | 5 | 149433596 | CSF1R | T | G | 99.1 | 100 | 99.6 | 0 | Novel | ST486/A549 |

**S1 Table. Genomic variants identified in the lung carcinoma cell line.** A total of sixteen (16) hotspot or novel variants were identified in parental A549 lung carcinoma cell line and ST486 Burkitt lymphoma cell lines (allele frequency ≥ 30%). Seven (7) variants showed enrichment in the A549 xMD sample, six (6) variants that were ST486-derived were suppressed in the A549 xMD sample. Two (2) variants were present in both cell lines and were unchanged. One variant, although present in both parent cell lines, was not detected in the xMD specimen.
