# Supplementary material for: High-Throughput Microdissection for Next-Generation Sequencing
Source: PLoS One. 2016 Mar 21;11(3):e0151775. doi: 10.1371/journal.pone.0151775 (PMC4801357; doi:10.1371/journal.pone.0151775)
Supplement: S2 Table — A total of sixteen (16) hotspot or novel variants were identified in parental UACC.62 melanoma cell line or ST486 Burkitt lymphoma cell line (allele frequency ≥ 30%). Five (5) variants showed enrichment in the UACC.62 xMD sample, nine (9) variants that were ST486-derived were suppressed in the UACC.62 xMD sample. Two (2) variants demonstrated equal frequencies in both cell lines. (DOCX) [file pone.0151775.s002.docx]

| Variant Status | Chromosome | Position | Gene | Ref | Variant | ST486 | UACC | UACC-M | UACC-X | Variant type | Source of Variant |
| --- | --- | --- | --- | --- | --- | --- | --- | --- | --- | --- | --- |
| xMD-Enriched | 7 | 140453136 | BRAF | A | T | 0 | 47.1 | 0 | 52.3 | Hotspot | UACC |
|  | 7 | 116411990 | MET | C | T | 0 | 49.3 | 0 | 30.3 | Hotspot | UACC |
|  | 9 | 21971116 | CDKN2A | G | A | 0 | 99.2 | 2.7 | 83.6 | Hotspot | UACC |
|  | 10 | 43613843 | RET | G | T | 50.9 | 100 | 51.3 | 91.8 | Novel | ST486/UACC |
|  | 10 | 43615633 | RET | C | G | 0 | 100 | 0 | 58.4 | Novel | UACC |
| xMD-Depleted | 2 | 212812097 | ERBB4 | T | C | 50 | 0 | 50.2 | 21.5 | Novel | ST486 |
|  | 5 | 112175240 | APC | G | C | 51.5 | 0 | 52.3 | 0 | Hotspot | ST486 |
|  | 5 | 112175770 | APC | G | A | 45.2 | 31.9 | 49 | 39.4 | Novel | ST486/UACC |
|  | 7 | 55249063 | EGFR | G | A | 100 | 52.4 | 100 | 48.4 | Novel | ST486/UACC |
|  | 11 | 534242 | HRAS | A | G | 49.8 | 0 | 46.8 | 10.8 | Hotspot | ST486 |
|  | 17 | 7578457 | TP53 | C | T | 51.7 | 0 | 47.9 | 15.9 | Hotspot | ST486 |
|  | 17 | 7577566 | TP53 | T | C | 48.9 | 0 | 49 | 12.8 | Hotspot | ST486 |
|  | 17 | 7579472 | TP53 | G | C | 57.8 | 0 | 59.4 | 7.1 | Novel | ST486 |
|  | 18 | 48586344 | SMAD4 | C | T | 47.1 | 0 | 46.1 | 11.4 | Novel | ST486 |
| Unchanged | 5 | 149433596 | CSF1R | T | G | 99.1 | 99.7 | 100 | 99.7 | Novel | ST486/UACC |
|  | 5 | 149433597 | CSF1R | G | A | 97.7 | 96.8 | 97.5 | 96.8 | Novel | ST486/UACC |

**S2 Table. Genomic variants identified in the melanoma cell line.** A total of sixteen (16) hotspot or novel variants were identified in parental UACC.62 melanoma cell line or ST486 Burkitt lymphoma cell line (allele frequency ≥ 30%). Five (5) variants showed enrichment in the UACC.62 xMD sample, nine (9) variants that were ST486-derived were suppressed in the UACC.62 xMD sample. Two (2) variants were present in both cell lines and were unchanged.
